# An unusual phenotype occurs in 15% of mismatch repair-deficient tumors and is associated with non-colorectal cancers and genetic syndromes

**DOI:** 10.1038/s41379-021-00918-3

**Published:** 2021-09-20

**Authors:** Marion Jaffrelot, Nadim Farés, Anne Cécile Brunac, Anne Pascale Laurenty, Marie Danjoux, David Grand, Samira Icher, Julie Meilleroux, Eliane Mery, Etienne Buscail, Charlotte Maulat, Christine Toulas, Pierre Vande Perre, Edith Chipoulet, Delphine Bonnet, Anne Staub, Rosine Guimbaud, Janick Selves

**Affiliations:** 1 Department of Digestive Oncology, Centre Hospitalier Universitaire (CHU), Toulouse, France; 2 Université Fédérale Toulouse Midi-Pyrénées, Université Toulouse III Paul Sabatier, INSERM, CRCT, 31330 Toulouse, France; 3 Department of Digestive Surgery, Centre Hospitalier Universitaire (CHU), Toulouse, France; 4 Department of Pathology, Institut Universitaire du Cancer-Oncopole de Toulouse; Centre Hospitalier Universitaire (CHU), Toulouse, France; 5 Department of Oncogenetics, Institut Universitaire du Cancer-Oncopole de Toulouse, Institut Claudius Regaud and Centre Hospitalier Universitaire (CHU), Toulouse, France

**Keywords:** Cancer screening, PCR-based techniques, Immunohistochemistry, Cancer screening

## Abstract

Immunohistochemistry (IHC) and/or MSI-PCR (microsatellite instability-polymerase chain reaction) tests are performed routinely to detect mismatch repair deficiency (MMR-D). Classical MMR-D tumors present a loss of MLH1/PMS2 or MSH2/MSH6 with MSI-High. Other profiles of MMR-D tumors have been described but have been rarely studied. In this study, we established a classification of unusual MMR-D tumors and determined their frequency and clinical impact. All MMR-D tumors identified between 2007 and 2017 were selected. Any profile besides the classical MMR-D phenotype was defined as unusual. For patients with unusual MMR-D tumors, IHC, and PCR data were reviewed, the tumor mutation burden (TMB) was evaluated and clinical and genetic features were collected. Of the 4948 cases of MMR testing, 3800 had both the available IHC and MSI-PCR results and 585 of these had MMR-D. After reviewing the IHC and PCR, 21% of the cases initially identified as unusual MMR-D were reclassified, which resulted in a final identification of 89 unusual MMR-D tumors (15%). Unusual MMR-D tumors were more often associated with non-CRC than classical MMR-D tumors. Unusual MMR-D tumors were classified into four sub-groups: i) isolated loss of PMS2 or MSH6, ii) classical loss of MLH1/PMS2 or MSH2/MSH6 without MSI, iii) four MMR proteins retained with MSI and, iv) complex loss of MMR proteins, with clinical characteristics for each sub-group. TMB-high or -intermediate was shown in 96% of the cancers studied (24/25), which confirmed MMR deficiency. Genetic syndromes were identified in 44.9% (40/89) and 21.4% (106/496) of patients with unusual and classical MMR-D tumors, respectively (*P* < 0.001). Five patients treated with an immune checkpoint inhibitor (ICI) had a prolonged clinical benefit. Our classification of unusual MMR-D phenotype helps to identify MMR deficiency. Unusual MMR-D phenotype occurs in 15% of MMR-D tumors. A high frequency of genetic syndromes was noted in these patients who could benefit from ICI.

## Introduction

Mismatch repair (MMR) proteins (MLH1, PMS2, MSH2, MSH6) are a major system of DNA repair that specifically repairs mismatches and small insertions/deletions that occur during cellular replication. MMR system deficiency (MMR-D) particularly affects microsatellites and results in alterations in their repeat length, named microsatellite instability (MSI), and in loss of expression of MMR proteins in tumors^1^. MMR-D is the hallmark of tumors associated with Lynch syndrome (LS), which is a cancer predisposition syndrome characterized by germline mutations in mismatch repair genes. MMR-D testing has traditionally been performed in patients with colorectal (CRC) or endometrial cancer (EC) to screen for LS-associated cancer predisposition^2,3^. MMR-D tumors also have a better prognosis and this phenotype can guide physicians in the use of adjuvant therapy for CRC or gastric cancer^4,5^. Finally, MMR-D has now been identified as a biomarker for response to immune checkpoint inhibitors (ICI) in metastatic diseases, regardless of the tumor origin^6^. The success of ICI in MMR-D tumors supports guidelines for testing MMR status in all advanced solid tumors. Consequently, testing for MMR-D is now incorporated in the routine oncological care of patients with advanced tumors across a broad spectrum of cancers^7^.

Traditionally, MMR-D identification has been based on MSI analysis by molecular biology, or immunohistochemical (IHC) analysis for MMR expression. In current practice, the terms MSI and MMR-D are used interchangeably to characterize MMR deficiency. The rule is to test the four main MMR proteins by immunohistochemistry: MLH1, MSH2, MSH6, and PMS2. Staining of the four proteins indicates an MMR-proficient tumor (MMR-P) and loss of protein staining indicates an MMR-D tumor^8^. Molecular biology is based on polymerase chain reaction (PCR) using five consensus microsatellites, and only tumors with an MSI-high (MSI-H) status with instability in two of the five microsatellites are classified as MMR-D^9^. Both methods are very sensitive with very good concordance; from 95% to nearly 99% for CRC^10,11^. The classical profile of an MMR-D tumor is the association of MSI-H with concomitant loss of the functional heterodimer MLH1/PMS2 or MSH2/MSH6. However, the MSI/MMR-D phenotype is less characterized in non-CRC, with recent studies showing a higher rate of discordance between microsatellite status and MMR immunophenotype^12–15^. Moreover, different types of MMR-D immunostaining from the classical MLH1/PMS2 or MSH2/MSH6 loss have also been described^16–18^. Consequently, the rising amount of MMR testing, particularly in non-CRC, has led to an increase in the number of unusual MMR-D cases. The origin and functional consequences of these unusual MMR-D profiles are not documented, nor has their clinical impact.

In this study, we first proposed a clear classification of unusual MMR-D tumor. We then sought to determine the frequency of unusual MMR-D tumors in a large cohort with consecutive MMR testing across a heterogeneous group of solid tumors, and to describe their clinical characteristics.

## Methods

### Study population

All consecutive MMR testing performed at Toulouse University Institute of Cancer-Oncopole between 1 January 2007 and 31 December 2017, were reviewed. Cases with both immunohistochemistry with the four MMR proteins and molecular biology data were selected. Patients with an MMR-D tumor constituted the population of interest. Patients gave their written consent for genetic analysis and the study was approved by our institutional board.

### Identification of unusual MMR-D tumors

Classical MMR-D tumors were defined as an MSI-H status associated with a loss of staining of both proteins of the functional heterodimer MLH1/PMS2 or MSH2/MSH6. Consequently, all other profiles of MMR-D tumors were defined as unusual MMR-D cases comprising: i) MSS (microsatellite stable) tumors associated with a loss of MMR protein immunostaining regardless of the immunostaining profile; ii) all MSI-Low tumors; and iii) MSI-High tumors with loss of staining different from the MLH1/PMS2 or MSH2/MSH6 heterodimer or with the retained expression of the four MMR proteins.

### MSI and IHC assays

MMR immunohistochemistry and MSI testing were performed according to standard procedures^19^. To evaluate MSI, the Pentaplex-PCR comprising five mononucleotide markers (BAT-25, BAT26, NR-21, NR-22, and NR-24) from the NCI panel was used^9,20^. For all tumors analyzed by PCR, at least 20% tumor cellularity was obtained.

Tumors were classified as MSI-H when at least two microsatellite markers showed instability, MSI-Low when only one marker showed instability and MSS when none of the markers showed instability^9^. For MSI-Low cases, if normal tissue was available, comparison with normal DNA was performed to eliminate polymorphism^21^.

Finally, all unusual MMR-D cases were verified. Slides of initial immunostaining and MSI data were reviewed by two pathologists (one junior and one senior) and if necessary, repeated when samples were available. MSI analyses were repeated in different FFPE blocks if necessary.

### NGS analysis

Twenty-six tumors were analyzed by next-generation sequencing (NGS) with the FoundationOne® test (F1CDx version) including an evaluation of microsatellite instability and tumor mutation burden (TMB). MSI tumors were defined by mutations in >30% of the microsatellite markers. TMB was classified into three sub-groups defined by the number of mutations per megabase (mut/Mb): TMB-high ≥ 20 mut/Mb, TMB-intermediate between 6 and 19 mut/Mb and TMB-low ≤ 5 mut/Mb.

### Clinical and biologic characteristics of unusual MMR-D

Clinical and biologic characteristics of MMR-D cases were retrospectively collected, including gender, age at diagnosis, histological type of tumor, stage, neoadjuvant treatment, treatment with ICI, and genetic investigations.

### Statistical analysis

Continuous and categorical variables were compared by using the two-tailed *t* test, the *Chi²* test (for parametric variables) and Fisher’s exact test (for nonparametric variables). *P* values < 0.05 were considered statistically significant.

## Results

### Identification of unusual MMR-D tumors

**Fig. 1:**
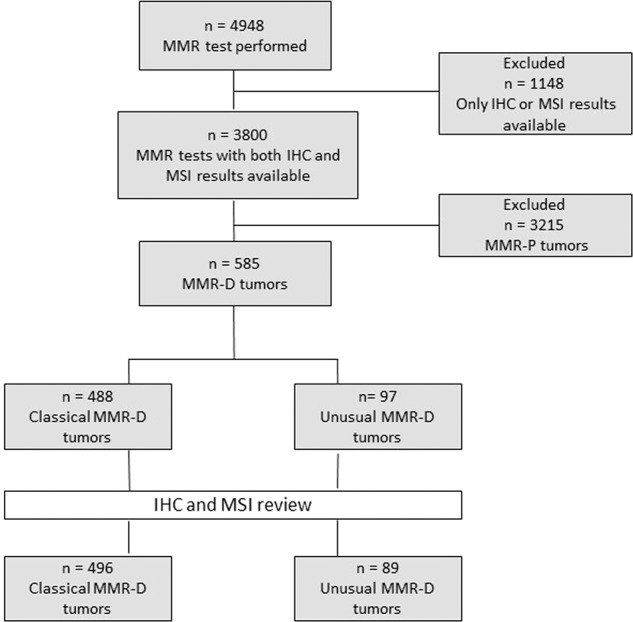
Flow chart. MMR: mismatch repair, IHC: immunohistochemistry, MSI: microsatellite instability, MMR-P: MMR proficient, MMR-D: MMR deficient.

Of 4948 tumors assessed for MMR testing, 3800 systematically had both IHC and MSI-PCR analysis. Among those cases evaluated by both methods, 585 were MMR-D (15.4%). Before reviewing the IHC and MSI, 488 of these 585 cases were categorized as classical MMR-D and 97 as unusual MMR-D tumors (Fig. 1).

### IHC and MSI review

**Fig. 2:**
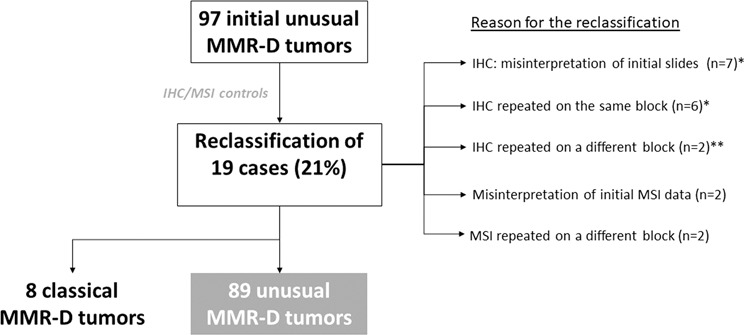
Identification of unusual MMR-D tumors after IHC and MSI review: *11/13 cases were clearly assigned to one category of loss of MMR proteins and 2/11 cases remained indeterminate for at least one MMR protein (PMS2). **: for one case, initial IHC was performed on biopsy with normal expression of MMR proteins but with an isolated loss of PMS2 on the surgical sample, and for the other case, initial IHC was performed on a polyp with normal expression of MMR protein but with PMS2/MLH1 loss on invasive carcinoma.

The initial IHC slides were reviewed for 90 unusual cases (seven samples not available). Immunostaining of seven cases was initially reported as not assessable for at least one of the four MMR proteins but the review of the slides enabled correct interpretation of the staining, except for two cases. Immunostaining of eight equivocal cases were repeated on the same block for six cases and on a different block for two cases (because the first was done on a polyp or on biopsy) using different antibodies and automat which enabled the interpretation of the staining (Fig. 2).

All the initial results of MSI testing were reviewed. Two MSI results were initially misinterpreted as MSS, while two of the five microsatellites were unstable. According to the current definition these cases were reclassified as MSI-High. Two cases initially classified as MSS tested on polyps were reclassified as MSI-High after microsatellite analysis of the corresponding carcinoma on a surgical resection specimen (Fig. 2).

After all the controls, the results for 19 cases were corrected: eight were reclassified as classical MMR-D tumors, and 11 were affirmed as unusual MMR-D but with a change of subgroup as defined below. Finally, 89 cases were identified as unusual MMR-D tumors, corresponding to 2.3% of the cohort with both IHC and MSI testing (89/3800) and to 15% of the MMR-D tumors (89/585) (Fig. 1).

### Classification of unusual MMR-D tumors

**Fig. 3:**
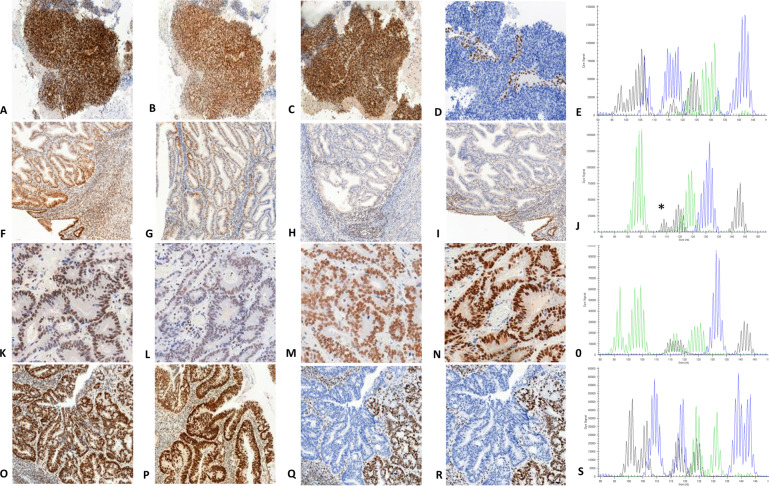
Representative images of MMR immunohistochemistry and microsatellite status assessed by MSI-PCR of each unusual MMR-D subgroup. Line 1 illustrates a group 1 unusual MMR-D: endometrial carcinoma with retained expression of MLH1, PMS2, and MSH2 (**A, B, C**,) isolated loss of MSH6 (**D**) and MSI-High (instability at the five markers of Pentaplex-PCR) (**E**). Line 2 shows an endometrial carcinoma in subgroup 2 with retained expression of MLH1 and PMS2 (**F, G**), loss of both MSH2 and MSH6 proteins (**H, I**), and MSI-Low in tumor DNA (**J**) at Bat 26 microsatellite only (asterisk) (comparison with normal DNA not shown). Line 3 shows a group 3 unusual MMR-D: a colon cancer with retained expression of the four MMR proteins (**K, L, M, N**) but MSI-High (instability at three of five markers of Pentaplex-PCR) (**O**). Line 4 illustrates a group 4 unusual MMR-D: colon cancer in a patient with LS with MSH6 germline mutation, retained expression of MLH1 and PMS2 (**O, P**) but a clonal loss of expression of MSH2 and MSH6 (**Q, R**) and MSI-High (instability at the five markers of Pentaplex-PCR) (**S**).

According to the IHC staining profile and microsatellite status, unusual MMR-D cases were then sub-divided into four groups: isolated loss of MSH6 or PMS2 regardless of the microsatellite status (group 1), classical loss of PMS2/MLH1 or MSH2/MSH6 but with MSI-Low or MSS (group 2), retained staining of the four MMR proteins with MSI-H or MSI-Low (group 3) and complex loss of MMR proteins regardless of the microsatellite status (group 4), which included concomitant loss of PMS2 and MSH6, loss of three or four proteins or clonal loss of MMR proteins (Table 1). These four sub-groups are illustrated in Fig. 3. According to the definition of discordant cases currently applied in the literature (loss of MMR proteins without MSI-H, or MSI-H without loss of MMR proteins), 31 of our 89 unusual MMR-D cases could be classified as discordant (10 in group 1, 16 in group 2, three in group 3, and two in group 4).

**Table 1:** Classification of unusual MMR-D tumors according to MMR IHC profiles and MSI status.

IHC\MSI	Group 1	Group 2	Group 3	Group 4	
	Isolated loss of PMS2 or MSH6	Classical loss of PMS2/MLH1 or MSH2/MSH6	MLH1/PMS2/ MSH2/MSH6 retained	Other types of MMR protein loss	All
**MSI-high**	43	Classical MMR-D	3	13	**59**
**MSI-low**	1	8	2	0	**11**
**MSS**	9	8	MMR-P	2	**19**
**All**	**53**	**16**	**5**	**15**	**89**

Tumors were sub-divided into four groups according to the IHC staining and MSI-PCR results. Group 1: isolated loss of PMS2 or MSH6 (with MSI-High, MSI-Low or MSS). Group 2: classical loss of PMS2/MLH1 or MSH2/MSH6 heterodimers (with MSI-Low or MSS, but not MSI-High since these cases correspond to classical MMR-D tumors). Group 3: retained staining of the four MMR proteins (with MSI-H or MSI-L but not MSS since these cases correspond to MMR-P tumors). Group 4: another type of MMR protein loss (with MSI-High, MSI-Low or MSS).

*MMR-D* mismatch repair-deficient tumor, *MMR-P* mismatch repair-proficient tumor, *IHC* immunohistochemistry, *MSI* microsatellite instability, MSI-High: instability of 2 or more microsatellites, MSI-Low: only one unstable microsatellite, MSS: stability of the 5 microsatellites.

### Clinical characteristics of patients with classical and unusual MMR-D tumors

The mean age at diagnosis, gender, cancer stage, histological type, and genetic syndrome of the two groups of patients (classical and unusual MMR-D) are compared in Table 2. Tumors were divided into four main categories according to histological types: CRC (491 cases), endometrial carcinoma (42 cases), non-colorectal gastrointestinal (GI) cancer (26 cases), and “other types of tumors” (26 cases). In the “other types of tumors” subgroup there were 12 sebaceous tumors, four urothelial carcinomas, one glioblastoma, six ovarian carcinomas, one sarcoma, one melanoma, and one neuroendocrine tumor. Non-colorectal GI cancers included three cholangiocarcinomas, five small bowel carcinomas, nine gastric carcinomas, two pancreatic carcinomas, four duodenal carcinomas, one hepatocellular carcinoma, and two peritoneal mucinous carcinomas. A genetic syndrome was diagnosed when a germline mutation known to drive a genetic predisposition to cancer was identified. Only five patients had neoadjuvant therapy: three patients with rectal cancer treated by radiochemotherapy before surgery, one patient with endometrial carcinoma treated with radiotherapy before surgery and one patient with pleomorphic sarcoma treated with radiochemotherapy before surgery (Table S1, online only).

**Table 2:** Clinical characteristics of patients with unusual and classical MMR-D tumors.

Characteristics	Unusual MMR-D (*n* = 89)	Typical MMR-D (*n* = 496)	*p*
**Mean age at diagnosis (years)**	57.5	59.7	0.15
**Gender (%)**			
Female	57.3 (*n* = 51)	56 (*n* = 278)	0.83
Male	43.7 (*n* = 38)	44 (*n* = 218)	–
**Histological type** (%)			
Colorectal carcinoma	67.4 (*n* = 60)	86.9 (*n* = 431)	<0.001
Non-colorectal GI carcinoma	9 (*n* = 8)	3.6 (*n* = 18)	–
Endometrial carcinoma	11.2 (*n* = 10)	6.5 (*n* = 32)	–
Other tumors	12.4 (*n* = 11)	3 (*n* = 15)	–
**Stage at diagnosis** (%)			
0 to III	85.4 (*n* = 76)	81 (*n* = 402)	0.74
IV	12.4 (*n* = 11)	15.7 (*n* = 78)	–
Missing data	2.2 (*n* = 2)	3.2 *(n* = 16)	–
**Genetic investigation** (%)			
Yes	60.7 (*n* = 54)	52.8 (*n* = 262)	0.17
No	39.3 (*n* = 35)	47.2 (*n* = 234)	–
Genetic syndrome identified (%)			
Yes	44.9 (*n* = 40)	21.4 (*n* = 106)	<0.001
Lynch Syndrome	42.7 (*n* = 38)	21.4 (*n* = 106)	–
POLE	1.1 (*n* = 1)	0	–
CMMR-D	1.1 (*n* = 1)	0	–
No	55.1 (*n* = 49)	78.6 (*n* = 390)	
Genetic syndrome identified among patients investigated (%)			
Yes	74.1 (*n* = 40)	40.5 (*n* = 106)	<0.001
No	25.9 (*n* = 14)	59.5 (*n* = 156)	–

*GI* gastrointestinal, *POLE* polymerase E deficiency syndrome, *CMMR-D* constitutional mismatch repair deficiency.

Other tumors included: for the 11 unusual MMR-D: 6 sebaceous tumors, 1 urothelial carcinoma, 1 glioblastoma, 2 ovarian carcinomas and 1 sarcoma and for the 15 classical MMR-D: 6 sebaceous tumors, 3 urothelial carcinomas, 1 melanoma, 4 ovarian carcinomas and 1 neuroendocrine tumor.

**Fig. 4:**
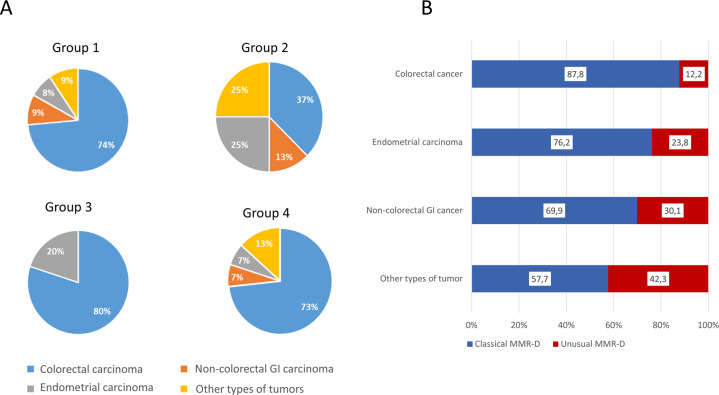
Distribution of the histological types among the different phenotypes of MMR-D. (**A**) Distribution of the histological types among the four sub-groups of unusual MMR-D tumors, and (**B**) proportion of classical and unusual MMR-D in the different histological types of the entire MMR-D cohort comprising 491 CRC, 42 endometrial carcinomas, 26 non-colorectal GI cancers, and 26 “other types of tumors” (results are in percentages). MMR-D: MMR deficiency, Non:colorectal GI carcinoma: noncolorectal gastrointestinal carcinoma.

The distribution of histological types was significantly different in the two groups (*P* *<* 0.001). The proportion of non-CRC was higher in the unusual (32.6 %) than in the classical MMR-D group (13.1%). Genetic investigations were carried out in a high proportion of cases, independently of the groups (respectively in 60.7 and 52.8%). Genetic syndromes were significantly more frequent in unusual (44.9%) than in classical MMR-D patients (21.4%) (*P* *<* 0.001). As expected, the main genetic syndrome identified in both groups was LS but two other syndromes were identified in the unusual MMR-D group: one polymerase E (POLE) deficiency and one CMMR-D (constitutional mismatch repair deficiency). The proportion of the different histological types among the four sub-groups of unusual MMR-D tumors is shown in Fig. 4A. A higher proportion of non-CRC cancers was observed in subgroup 2 (63% of non-CRC), corresponding to classical loss of PMS2/MLH1 or MSH2/MSH6 but with MSI-Low or MSS. Regarding the distribution of unusual MMR-D among the histological types, the highest frequency of unusual MMR-D was observed for “other types of tumors” (42.3%), followed by non-colorectal GI cancers and endometrial carcinomas (30.1 and 23.8%, respectively) whereas only 12.2% of CRC had unusual MMR-D (Fig. 4B).

Characteristics of the four sub-groups of unusual MMR-D are described in Table 3. Group 1 (isolated loss of PMS2 or MSH6) represented the largest subgroup (60%), with a lack of microsatellite instability in 19% of the cases (1 MSI-Low and 9 MSS) and corresponded mainly to CRC (73.5%). Geneticist consulting, performed in 36 cases, led to the identification of 24 genetic syndromes (45.3%). One patient had a rectal carcinoma treated with radiochemotherapy before surgery. He had an isolated loss of MSH6 with MSI, TMB-High with 108 mut/Mb, and somatic mutation of MSH6.

**Table 3:** Characteristics of the four unusual MMR-D sub-groups.

	MSI-High	MSI-Low*	MSS	All
**Group 1 (Isolated loss of PMS2 or MSH6)**	**43**	**1**	**9**	**53**
Histologic type				
CRC	35	1	3	39
GI cancer, no CRC	4	0	1	5
Endometrial cancer	3	0	1	4
Other types	1	0	4	5
Protein Loss				
PMS2	17	0	4	21
MSH6	26	1	5	32
Genetics				
Investigation performed	28	1	7	36
Lynch syndrome (PMS2)	8	0	2	10
Lynch syndrome (MSH6)	11	0	1	12
CMMR-D	0	0	1	1
POLE	0	0	1	1
% genetic syndromes in the group				45.3%
Lynch/isolated loss of PMS2				47.6%
Lynch/isolated loss of MSH6				37.5%
**Group 2 (Loss of PMS2/MLH1 or MSH2/MSH6)**		**8**	**8**	**16**
Histological Type				
CRC	–	3	3	6
GI cancer, no CRC	–	0	2	2
Endometrial cancer	–	3	1	4
Other types	–	2	2	4
Heterodimer Loss				
MLH1/PMS2	–	5	6	11
MSH2/MSH6	–	3	2	5
Genetics				
Investigation performed	–	3	3	6
Lynch syndrome		3	3	6
% Lynch syndrome in the group				37.5%
**Group 3 (Four MMR proteins retained)**	**3**	**2**	**0**	**5**
Histological type				
CRC	3	1**	–	4
GI cancer, no CRC	0	0	–	0
Endometrial cancer	0	1	–	1
Other types	0	0	–	0
Genetics				
Investigation performed	2	0	–	2
Lynch syndrome	2	0	–	2
% Lynch syndrome in the group				40%
**Group 4 (Complex loss of MMR proteins)**	**13**	**0**	**2**	**15**
Histological Type				
CRC	11	–	–	11
GI cancer, no CRC	1	–	–	1
Endometrial cancer	1	–	–	1
Other types	–	–	2	2
Protein Loss				
4 MMR proteins	4	–	1	5
2 proteins, no heterodimers	2	–	–	2
3 MMR proteins	3	–	–	3
Heterogeneous staining	3	–	–	3
NI***	1		1	2
Genetics				
Investigation performed	9	–	2	11
Lynch syndrome	6	–	2	8
% Lynch syndrome in the group				53.3%

*CRC* colorectal carcinoma, *GI* gastrointestinal, *POLE* polymerase E deficiency syndrome, *CMMR-D* constitutional mismatch repair deficiency, *NI* not informative.

For each subgroup, histological type, MMR immunohistochemistry profile, and genetic data are presented, as well as MSI status (MSI-High, MSI-Low, and MSS).

*diagnosis retained after comparison with normal DNA for six cases, without normal DNA available for four cases but with MMR protein loss.

**for this MSI-low CRC, a polymorphism cannot be excluded because comparison with normal DNA was not performed (normal DNA was not available).

***two non-informative tumors after IHC controls, one case: MSH6: background staining, PMS2: no internal control, staining of MLH1 and MSH2 retained and one case: PMS2: no internal control, staining of the three others proteins retained.

Group 2 (loss of MLH1/PMS2 or MSH2/MSH6 but without MSI-H) represented 18% of the unusual MMR-D. This subgroup had a high percentage of non-CRC tumors (63%). Only six patients had geneticist consulting but they all had LS. One patient had an endometrial carcinoma treated with radiotherapy before surgery, the tumor was MSS with a loss of MLH1 and PMS2.

Group 3 (four MMR proteins retained with MSI-Low or MSI-H) consisted of only five patients (5.6% of the unusual MMR-D). Only two patients had geneticist consulting and both had LS. One patient had a rectal carcinoma treated with radiochemotherapy before surgery. He had LS with PMS2 germinal mutation and his tumor was MSI with retained expression of the four MMR proteins.

Group 4 (complex loss of MMR protein) was mostly associated with MSI-H and only two of the 15 cases were MSS. This subgroup consisted of 73.3% CRC. Geneticist consulting performed for 11 patients led to the identification of eight LS (53.3%). Two patients had radiochemotherapy before surgery: one had rectal cancer and LS with PMS2 germinal mutation, MSI, and clonal loss of PMS2 in the tumor; the other had a pleomorphic sarcoma and LS with germinal mutation of MLH1. His tumor showed a loss of the four MMR proteins but was MSS.

**Fig. 5:**
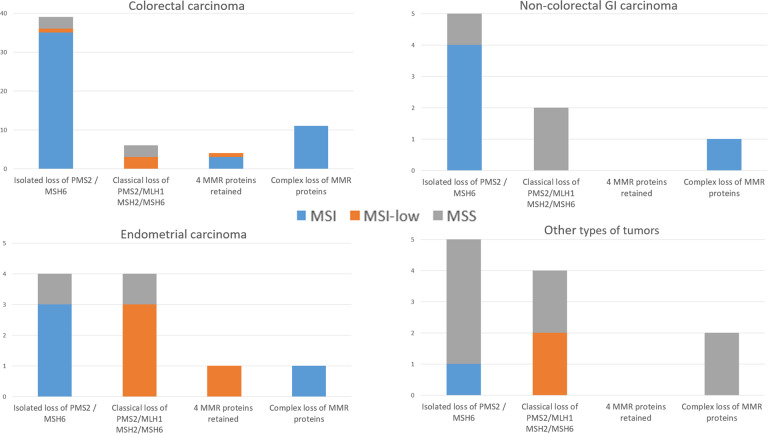
Distribution of each group of unusual MMR-D tumors among the histological types. Non-colorectal GI carcinoma: non-colorectal gastrointestinal carcinoma, MSI: microsatellite instability, MSS: microsatellite stable.

The distribution of each subgroup of unusual MMR-D tumors (groups 1, 2, 3, and 4) among the four categories of histological types is shown in Fig. 5.

Unusual MMR-D CRC (60 cases): as expected, the largest subgroup was group 1 (isolated loss of PMS2 or MSH6) but the three other sub-groups were also present in CRC. True discordant cases were rare in CRC (13/60 cases), among them four were MSI-low.

Unusual MMR-D endometrial carcinoma (10 cases): the four sub-groups were present with predominance of group 1 (isolated loss of PMS2 or MSH6) and group 2 (classical loss of MLH1/PMS2 or MSH2/MSH6 without MSI). MSI-Low was frequent in sub-groups 2 and 3 (4 MMR proteins retained). Half of the cases (5/10) were discordant, mainly due to MSI-low (3/5).

Unusual MMR-D non-colorectal GI cancer (eight cases): the largest subgroup was group 1 (isolated loss of PMS2 or MSH6) and there were no cases in group 3 (retained four MMR proteins) nor MSI-low tumors. Three cases were discordant.

Unusual MMR-D “other types of tumors” (13 cases): Groups 1 (isolated loss of PMS2 or MSH6), 2 (classical loss of MLH1/PMS2 or MSH2/MSH6 without MSI), and 4 (complex loss of MMR proteins) were present but there was no case in group 3 (retained 4 MMR proteins). Two group 2 cases were MSI-low tumors. Ten cases were discordant.

### NGS analysis

**Fig. 6:**
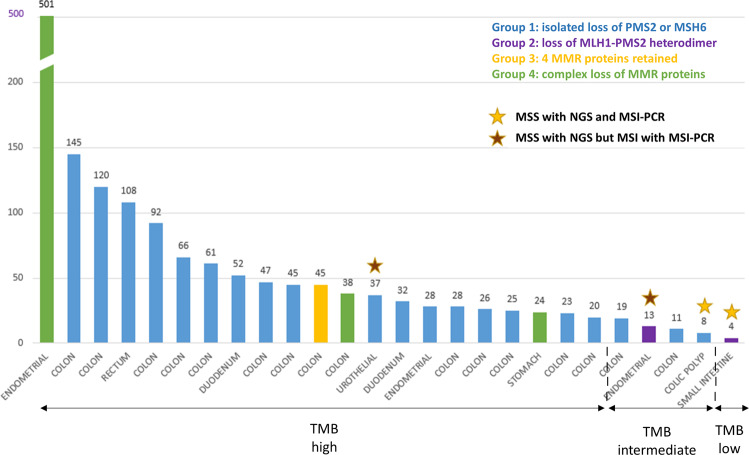
NGS analysis. Tumor mutational burden (TMB) is reported as mutations per megabase (mut/Mb) in the *y*-axis. Tumor types are indicated on the *x*-axis. The TMB value is mentioned above each graph bar. Tumors were divided into three categories according to TMB value: TMB-high: ≥20 mut/Mb, TMB-intermediate: between 6 and 19 mut/Mb and TMB-low: ≤5 mut/Mb. Even after excluding the only ultra-mutated tumor (501 mut/Mb) corresponding to LS associated with a somatic PolD1 mutation, the range of TMB values was highly variable, ranging from 4 to 145 mut/Mb. All cases, except for four cases, were MSI by NGS analysis. Stars above the graph bars indicate MSS (microsatellite stable) tumors; yellow stars represent MSS tumors both with NGS and MSI-PCR and brown stars indicate MSS tumors with NGS but MSI-H with MSI-PCR.

Of the 89 unusual MMR-D tumors, 26 were analyzed with the FoundationOne® test and were mainly tumors from group 1 (20 cases). Twenty-one tumors were TMB-High, four were TMB-intermediate, one was TMB-low and twenty-two were MSI (Fig. 6). Concordance between the MSI-PCR and NGS results for microsatellite status, TMB data, IHC patterns, and genetic results are presented in Supplemental Table S1, (online only). All of the 21 TMB-high tumors were classified as MSI by NGS except for one “false-negative” case: a urothelial carcinoma in LS with isolated loss of MSH6, MSI with MSI-PCR, and 37 mut/Mb. Among the four TMB-intermediate tumors, one was MSS but based on a colon polyp analysis, two cases were colon cancers with MSI, both with NGS and MSI-PCR and the remaining case was an endometrial carcinoma, MSI-Low by MSI-PCR and MSS by NGS.

### Impact of the type of MMR protein inactivation on unusual MMR-D phenotype

The impact of inactivation of each MMR protein (MLH1, PMS2, MSH2, or MSH6) on the immunostaining profile and microsatellite status is presented in Supplemental Table S2 (online only). Inactivation of an MMR protein was defined by the identification of a germinal or somatic mutation of the corresponding gene (based on genetic or tumor NGS analysis or on MLH1 promotor methylation in the tumor). We defined a second category of tumors with MMR protein inactivation which corresponded to tumors with loss of expression of one MMR protein but with no mutation identified (neither germinal nor somatic) and no genetic investigations. The most frequently inactivated protein in the group of unusual MMR-D tumors was MSH6 (34 cases), followed by PMS2 (25 cases), MLH1 (15 cases) and MSH2 (only eight cases). Regarding PMS2 and MSH6 inactivation, the performance of Pentaplex-PCR in MSI-High identification was only 80% (20/25) and 79.4% (27/34) respectively. This performance was better in the subgroup of patients with an identified genetic cause: 84% and 90% respectively for PMS2 and MSH6 mutation. Conversely, IHC showed loss of staining of the corresponding mutated protein in 95% (18/19) and 100% (20/20) of the cases with PMS2 and MSH6 mutation respectively. No case with PMS2 mutation showed concomitant loss of MLH1/PMS2 staining. Similarly, no case with MSH6 mutation showed concomitant loss of MSH6/MSH2 staining. Regarding MLH1 and MSH2 protein inactivation, the performance of each method (IHC and Pentaplex-PCR) in MMR deficiency identification could not be correctly evaluated because of the low number of cases in each category and a high proportion of cases without genetic investigations.

### Characteristics of patients treated with anti-PD1/PD-L1 immunotherapy

Of 89 unusual MMR-D patients, 17 had a metastatic disease including five treated with ICI. Two had a complete response (group 1), one had a stable disease (group 1) and two patients (group 2) had a partial response for 7 and 10 months respectively before progression. Their characteristics are summarized in Table 4.

**Table 4:** Characteristics of patients treated with immunotherapy.

Type of cancer	Metastasis	IHC	MSI-PCR	Type and period of immunotherapy	Line of treatment	Response	Genetic syndrome
CRC	Liver peritoneum	Isolated loss of MSH6	MSI-H	PEMBROLIZUMAB (12/2015 - 12/2017)	6th	Complete response	Not investigated
Cholangiocarcinoma	Peritoneum	MLH1/PMS2 loss	MSS	PEMBROLIZUMAB (03/2016 - 11/2016)	2nd	Partial response for 7 months, then progression and death	Lynch (MLH1)
Gastric carcinoma	Lymph nodes, Liver, adrenal gland	Isolated loss of MSH6	MSS	NIVOLUMAB + anti-LAG3 (11/2016 - 04/2018)	2nd	Complete response	Not identified
CRC	Liver peritoneum	MLH1/PMS2 loss	MSS	PEMBROLIZUMAB (12/2017 - 10/2018)	2nd	Partial response for 10 months, then progression	Not investigated
Peritoneal mucinous carcinoma	Peritoneum Lung	Isolated loss of MSH2	MSI-H	PEMBROLIZUMAB (12/2017 - still treated)	1st	Stable disease	Lynch (MSH2)

*CRC* colorectal carcinoma, *IHC* immunohistochemistry, *MSI-H* MSI High.

Data for MMR immunostaining, MSI-PCR, NGS, MLH1 promotor methylation, genetics, and relevant treatments for the entire cohort of 89 unusual MMR-D cases are presented in Supplemental Table S1 (online only).

## Discussion

Systematic assessment of MMR deficiency with both the IHC and MSI-PCR, spanning 3800 tumors of different histology, shows that 15% of MMR-D tumors present an unusual phenotype. To our knowledge, this is the largest analysis described in the literature and the first to propose an exhaustive classification of unusual MMR-D tumors. Unusual MMR-D phenotype includes not only discordant phenotypes (loss of MMR proteins without MSI-H or MSI-H without loss of MMR protein), amounting to a total of 31 cases, but all phenotypes different from the classical MMR-D phenotype characterized by MSI-H with loss of MLH1/PMS2 or MSH2/MSH6. This exhaustive definition enabled the inclusion of the MSI-Low category, for which the diagnostic value for MMR deficiency is controversial^22,23^, all immunostaining profiles different from the loss of MLH1/PMS2 or MSH2/MSH6 and all cases with a discordance between the IHC and MSI-PCR results.

Because IHC helps to identify the cause of MMR deficiency, we also suggest classifying unusual MMR-D tumors into four sub-groups according to the IHC profile; group 1: isolated loss of PMS2 or MSH6 regardless of the microsatellite status, group 2: classical loss of MLH1/PMS2 or MSH2/MSH6 but with MSI-Low or MSS, group 3: four MMR proteins retained with MSI-H or MSI-Low and group 4: complex loss of MMR protein staining regardless of the microsatellite status. We choose to consider the isolated loss of PMS2 or MSH6 as an unusual MMR-D phenotype although those cases are usually assimilated with classical MMR-D tumors^18,24^. In fact, this subgroup is poorly characterized in the literature, mainly in case reports. Major unresolved questions are the functional consequences of isolated loss of PMS2 or MSH6 on MMR system efficiency and their significance for the diagnosis of LS. Moreover, the rate of discordant phenotypes in this subgroup of tumors has not been widely examined.

The high frequency of unusual IHC/PCR results (15% of MMR-D tumors, but only 2.6% of MMR testing) may be explained by our exhaustive definition of unusual MMR-D phenotype, which included isolated loss of MSH6 or PMS2 with MSI-H. Those cases (*n* = 43) are unusual according to our definition but not discordant between the IHC and PCR.

A systematic review and/or control of IHC and MSI testing data reinforces the robustness of our results. This allowed the initial results for 21% of the cases to be changed, mainly IHC. Eight cases were reclassified as classical MMR-D and the remaining 89 cases were “true” unusual MMR-D. This reclassification was partly due to the improvement in IHC assay performance over the past 10 years, but mainly to the application of the new rules for MMR immunostaining interpretation when reviewing. We applied the decision tool recently validated to interpret MMR immunostaining of endometrial and ovarian carcinomas^25,26^. This is a very useful tool in the analysis of faint or equivocal staining. We also took into account some rare and “complex” types of MMR protein losses, including clonal loss of staining^27^, loss of two proteins different from functional heterodimers^28^, and loss of three or four MMR proteins^29^. Only four microsatellite statuses were reclassified. This very low rate of mistakes for MSI analysis confirmed the accuracy of MSI-PCR when the technical recommendations, which were implemented in this study, are respected: at least 20% tumor-cellularity for MSI analysis, strict application of the MSI-Low definition, and molecular analysis only in invasive carcinoma^9,30,31^.

We showed that unusual MMR-D phenotypes were more often associated with non-CRC than classical MMR-D tumors, reaching 23.8% of MMR-D endometrial carcinomas, 30.1% of non-colorectal GI cancers, and 42.3% of other types of tumors. This was especially pronounced for group 2 (loss of PMS2/MLH1 or MSH2/MSH6 without MSI), which included only 37% of CRC compared to 86.9% of CRC in classical MMR-D. More precisely, PCR failed to detect MSI-H in tumor with MMR protein loss in only 2% of all MMR-D CRC (10/487), but in 12.2% of endometrial carcinomas (5/41), 11.5% of noncolorectal GI cancers (3/26), and 38.5% of “other types of tumors” (10/26). This supports the notion of a lack of sensitivity of MSI-PCR for non-CRCs, which is also documented for gliomas, prostate, and urothelial carcinomas and sebaceous tumors^32–35^. Conversely, lack of MSI-PCR to identify MMR deficiency in endometrial carcinoma is usually reported as more limited (6%) than observed in our study^12^. MSI-PCR and IHC are usually reported as very sensitive and specific methods to detect MMR deficiency in gastric carcinoma with a good concordance between the two methods, but have rarely been evaluated in other GI cancers^36^. ESMO guidelines for immunotherapy recently recommended the use of MMR immunohistochemistry as the primary method for MSI testing in any cancer belonging to the LS spectrum (colorectal, endometrial, small intestine, urothelial, gliomas/glioblastomas, and sebaceous gland) and to move to MSI-PCR whenever the IHC interpretation is doubtful and as a confirmatory test in case of isolated loss of PMS2 or MSH6^37,38^. However, the main limitation of this two-step strategy is the risk of missing rare MMR-D tumors with retained MMR protein expression, which can account for up to 6% of MMR-D tumors according to recent work by Hechtman et al.^39^. Through a rigorous technique and interpretation of IHC, we have shown that these cases (group 3) represent less than 1% (5/585) of all MMR-D tumors, which validates IHC as the primary method to detect MMR deficiency in a broad spectrum of tumors. Nevertheless, our good results are based on practice in a tertiary center with a large volume of activity and IHC performance may be lower in routine practice. This underlines the need for good calibration of the IHC technique, as well as training in MMR staining interpretation^40^. The role of MSI-PCR as a second test to confirm MMR-D tumors still remains important^41^. It can help to confirm MMR-deficient tumors without the use of expensive and scarce techniques, such as NGS. NGS-based MSI testing has the potential to become the method of choice for all tumor types that do not belong to the LS spectrum^7,42,43^. We were able to test the contribution of NGS for MSI detection in 26 cases with the FoundationOne® test. In this limited number of cases, the performance of NGS to detect MSI tumors seems to be inferior to MSI-PCR (84 vs. 92%, respectively), suggesting that the panel of microsatellites used in this NGS testing was sub-optimal for detecting unusual MMR-D tumors^44,45^. This result supports the notion that all MSI-NGS assays should be validated against MMR IHC or MSI by PCR and should show its equivalency before application in laboratories. NGS also enabled the evaluation of TMB. Twenty-four of the twenty-five unusual MMR-D invasive cancers were TMB-high or -intermediate, which confirmed MMR deficiency.

Finally, our work supports the ESMO guideline strategy for starting with IHC, but the second step of MSI-PCR should be improved for non-CRC by using more appropriate microsatellite panels. We also think that the place of NGS in discordant cases is very promising, rather for TMB evaluation than for MSI evaluation, but these two approaches should be robustly validated for each tumor type before use in routine practice. Therefore, at present we cannot recommend any particular NGS method.

Our study provides further information about the impact of the type of inactivated protein on unusual MMR-D profile. The most frequently inactivated proteins in the group of unusual MMR-D tumors are MSH6 (34 cases) and PMS2 (25 cases). Our study showed a lower performance of Pentaplex-PCR than IHC in the detection of PMS2 and MSH6 inactivation. This is expected for MSH6-deficient tumors which are reported to be associated with a lower proportion of unstable markers and a smaller size of allelic shifts resulting in some false-negative results. However, this phenomenon has not been described for PMS2. We also showed that IHC is very effective in the detection of these two mutated proteins (95% sensitivity for PMS2 and 100% for MSH6). Our results are important considering the conflicting results reported on the performance of each method in these two groups of tumors. Regarding MSH6 loss due to a constitutional mutation, You et al reported that Pentaplex-PCR had very good sensitivity (97%) in MSI detection in 29 cases of tumors of various origins (mainly CRC), whereas another studies reported lower Pentaplex-PCR sensitivity (84%) in 19 tumors of various origins^24,46^. Regarding PMS2 loss, Van der Klift et al. showed that PCR had a sensitivity of only 86% in MSI detection in a series of 130 PMS2 pathogenic mutations^47^. Conversely, in a recent study of 136 patients with pathogenic PMS2 mutation, a very good performance was reported for Pentaplex-PCR (96%) in MSI detection^48^. There were a few cases of unusual MMR-D due to MLH1 or MSH2 inactivation in our study (23 cases in comparison to 496 cases in the classical MMR-D group), which confirms that discordance between IHC and Pentaplex-PCR is a rare event for these two proteins.

Our study shows that unusual MMR-D tumors are significantly more often associated with a genetic syndrome than classical MMR-D tumors. We identified twice as many genetic syndromes (44.9%) in the unusual MMR-D population as in the classical MMR-D population (21.4%) despite the high volume of CRC in our cohort, which classically contain a high proportion of sporadic MMR-D cancers due to hypermethylation of the *MLH1* promotor.

Two sub-groups had a particularly high level of genetic predisposition. As expected, the first was isolated loss of PMS2 or MSH6 (group 1), with 45.2% genetic syndromes, mainly LS but also one PolE deficiency and one CMMR-D. For isolated PMS2 loss, the prevalence of LS (47.6%) was very close to that reported in previous studies for CRC and endometrial carcinoma, varying from 35% to 45%^16,49^. Regarding isolated loss of MSH6, the prevalence of LS is less clear in the literature since many cases have been reported to be associated with somatic inactivation of MSH6, for example, secondary to neoadjuvant therapy^17,50^. In our cohort, only one patient out of 5 who received neoadjuvant treatment had an isolated loss of MSH6, but had MSI, with a TMB-High (108 mut/Mb). Our study is the first to investigate the prevalence of LS in patients with an isolated loss of MSH6, which was evaluated at 37.5%. The second subgroup, which had a high percentage of genetic syndromes (53.3%), was subgroup 4 (complex loss of MMR proteins). Different mechanisms could explain the immunostaining profiles of this very heterogeneous group: double somatic inactivation affecting both dominant MMR genes or a combination of constitutional mutation and sporadic alteration^13,51,52^. A purely somatic inactivation is the main cause of endometrial carcinoma with complex loss of MMR proteins^12^. Conversely, in our study, such complex immunostaining profiles were mainly due to LS, probably linked to a high proportion of CRC in this subgroup (73%).

One of the urgent questions to be addressed is whether unusual MMR-D patients could benefit from an immune checkpoint inhibitor (ICI). In our cohort, only five patients were treated with ICI because of their good prognosis (19% of the patients with metastatic disease) but also due to limited access to ICI in Europe. Our results indicated a prolonged clinical benefit for these five patients, which confirms the behavior of these cases as MMR-D and highlights the importance of not excluding them from ICI therapy.

Finally, the retrospective and monocentric design of our study, which was the main limitation, was compensated by a systematic double-review of all unusual MMR-D cases. Moreover, this design limited the ability to obtain genetic data and to perform NGS analysis for all our unusual MMR-D patients. One criticism of our study may be to classify isolated loss of PMS2 or MSH6 as unusual MMR-D. Several results of our work support identifying these tumors as unusual MMR-D phenotypes: nearly one in five tumors of this subgroup (19%) had a discordant result with MSI testing (a very high rate compared to 3% (16/512) in the group with MLH1/PMS2 and MSH2/MSH6 loss), their value for LS diagnosis is not as high as expected, mainly for isolated MSH6 loss. Further studies are needed to determine the origin of MMR deficiency in this subgroup of tumors as well as their sensitivity to ICI.

In conclusion, our study showed that 15% of MMR-D tumors determined by both IHC and MSI-PCR harbor unusual MMR-D phenotypes and are more frequent in non-CRC. Their identification could be difficult (21% of the cases were initially misclassified) and needs rigorous techniques and interpretation of results. Ours is the first proposal for a classification of these unusual phenotypes into four sub-groups which is useful for the diagnosis of MMR deficiency. Unusual MMR-D tumors, including discordant profiles as well as more complex patterns, are associated with a high frequency of genetic syndromes (45%) and are TMB-high or -intermediate. Consequently, these patients should benefit from genetic investigation and should not be excluded for ICI therapy.

## Supplementary information

Supplementary informations
