# Supplementary material for: An unusual phenotype occurs in 15% of mismatch repair-deficient tumors and is associated with non-colorectal cancers and genetic syndromes
Source: Mod Pathol. 2021 Sep 20;35(3):427–37. doi: 10.1038/s41379-021-00918-3 (PMC8860743; doi:10.1038/s41379-021-00918-3)
Supplement: Supplementary file 1 — Supplementary informations [file 41379_2021_918_MOESM1_ESM.docx]

**SUPPLEMENTARY INFORMATIONS (online only).**

**Table S1** (**online only)**: Histological types, MMR immunostaining, MSI-PCR, NGS, MLH1 promotor methylation, genetics and relevant treatment for the entire cohort of 89 unusual MMR-D cases.

| **Histological type** | **MMR Immunostaining** | **MSI-PCR** | **Atypical-MMR-D sub-group** | **NGS results:**  **MSI status**  **TMB**  **MMR mutation**  **MLH1 promotor methylation** | **Genetics** | **Neoadjuvant treatment or ICI** |
| --- | --- | --- | --- | --- | --- | --- |
| Colon carcinoma | Clonal loss PMS2 MLH1 | MSI (5) | Group 4 | MSI High  TMB High: 38  PMS2 | No LS (no PMS2 germline mutation) | - |
| Colon carcinoma | Isolated loss MSH6 | MSI (5) | Group 1 | MSI High  TMB High: 145  MSH6 | No genetic investigation (refusal) | - |
| Colon carcinoma | Isolated loss MSH6 | MSI (5) | Group 1 | MSI High  TMB High: 120  MSH6 | No genetic investigation (refusal) | - |
| Rectal carcinoma | Isolated loss MSH6 | MSI (5) | Group 1 | MSI High  TMB High: 108  MSH6 | No genetic investigation | RCT |
| Colon carcinoma | Isolated loss MSH6 | MSI (4) | Group 1 | MSI High  TMB High: 92  MSH6 | LS (MSH6) | - |
| Colon carcinoma | Isolated loss MSH6 | MSI (5) | Group 1 | MSI High  TMB High: 66  MSH6 | No LS (no MSH6 germline mutation) | - |
| Colon carcinoma | Isolated loss MSH6 | MSI (5) | Group 1 | MSI High  TMB High: 61  MSH6 | No genetic investigation | - |
| Colon carcinoma | Isolated loss PMS2 | MSI (5) | Group 1 | MSI High  TMB High: 28  PMS2 + MSH6 | Genetic analysis on going | - |
| Colon carcinoma | Isolated loss MSH6 | MSI (5) | Group 1 | MSI High  TMB High: 47  MSH6 | No LS (no MSH6 germline mutation) | - |
| Colon carcinoma | Retained staining of 4 proteins | MSI (3) | Group 3 | MSI High  TMB High: 45  MLH1 + PMS2 | No genetic investigation | - |
| Colon carcinoma | Isolated loss MSH6 | MSI (3) | Group 1 | MSI High  TMB High: 45  MSH6 | LS (MSH6) | - |
| Colon carcinoma | Isolated loss MSH6 | MSI (4) | Group 1 | MSI High  TMB High: 26  MSH6 | No genetic investigation | - |
| Colon carcinoma | Isolated loss PMS2 | MSI (5) | Group 1 | MSI High  TMB High: 25  PMS2 + MSH3 | LS (PMS2) | - |
| Colon carcinoma | Isolated loss PMS2 | MSI (5) | Group 1 | MSI High  TMB High: 23  PMS2 | No genetic investigation | - |
| Colon carcinoma | Isolated loss PMS2 | MSI (4) | Group 1 | MSI High  TMB High: 20  PMS2 | LS (PMS2) | - |
| Colon carcinoma | Isolated loss PMS2 | MSI (5) | Group 1 | MSI High  TMB intermediate: 19  PMS2 | No Lynch (no PMS2 germline mutation) | - |
| Colon carcinoma | Isolated loss PMS2 | MSI (5) | Group 1 | MSI High  TMB intermediate.: 11  PMS2 et MSH6 | LS (PMS2) | - |
| Colorectal polyp | Isolated loss PMS2 | MSS | Group 1 | MSS  TMB intermediate: 8  PMS2 +MLH1 | CMMR-D  (PMS2) | - |
| Colon carcinoma | Loss MLH1/PMS2 | MSI Low (1) | Group 2 | No NGS  No methylation analysis | No genetic investigation | - |
| Colon carcinoma | Retained MSH2/MSH6  /MLH1, PMS2 NI | MSI (5) | Group 4 | - | LS (MLH1) | - |
| Colon carcinoma | Isolated loss MSH6 | MSS | Group 1 | - | POL-E | - |
| Rectal carcinoma | Isolated loss MSH6 | MSS | Group 1 | - | No genetic investigation | - |
| Colon carcinoma | Retained MSH2/PMS2  /MLH1, MSH6 NI | MSI (5) | Group 4 | - | LS (MSH6) | - |
| Rectal carcinoma | Isolated loss MSH6 | MSI Low (1) | Group 1 | - | Genetic analysis on going | - |
| Colon carcinoma | Clonal loss MLH1/PMS2, retained MSH2, MSH6 NI | MSI (4) | Group 4 | - | LS (MLH1) | - |
| Colon carcinoma | Isolated loss MSH6 | MSI (3) | Group 1 | - | LS (MSH6) | - |
| Colon carcinoma | Isolated loss PMS2 | MSI (5) | Group 1 | - | LS (PMS2) | - |
| Colon carcinoma | Isolated loss MSH6 | MSI (5) | Group 1 | - | LS (MSH6) | - |
| Colon carcinoma | Isolated loss MSH6 | MSI (3) | Group 1 | - | No genetic investigation | - |
| Rectal carcinoma | Retained MSH2/MSH6 /MLH1, clonal loss PMS2 | MSI (5) | Group 4 | - | LS (PMS2) | RCT |
| Colon carcinoma | Isolated loss MHS6 | MSI (5) | Group 1 | - | No genetic investigation | ICI |
| Colon carcinoma | Isolated loss PMS2 | MSI (5) | Group 1 | - | LS (PMS2) | - |
| Rectal carcinoma | Loss MLH1/PMS2 | MSI Low (1) | Group 2 | No NGS  No methylation analysis | No genetic investigation | - |
| Rectal carcinoma | Isolated loss MHS6 | MSI (3) | Group 1 | - | Genetic analysis on going | - |
| Colon carcinoma | Loss MSH2/MSH6 | MSS | Group 2 | - | No genetic investigation | - |
| Colon carcinoma | Retained MLH1/MSH2 /MSH6, PMS2 NI | MSI (5) | Group 4 | - | LS (PMS2) | - |
| Rectum carcinoma | Retained 4 proteins | MSI (5) | Group 3 | - | LS (MSH2) | RCT |
| Colon carcinoma | Isolated loss MSH6 | MSI (5) | Group 1 | - | No genetic investigation | - |
| Colon carcinoma | Retained MLH1/MSH2, MSH6/PMS2 NI | MSI (4) | Group 4 | - | No genetic investigation | - |
| Colon carcinoma | Isolated loss MSH6 | MSI (5) | Group 1 | - | No genetic investigation | - |
| Colon carcinoma | Isolated loss MSH6 | MSI (5) | Group 1 | - | LS (MSH6) | - |
| Rectal carcinoma | Isolated loss MSH6 | MSI (2) | Group 1 | - | No genetic investigation | - |
| Colon carcinoma | Isolated loss PMS2 | MSI (5) | Group 1 | - | No LS | - |
| Colon carcinoma | Isolated loss MSH6 | MSI (5) | Group 1 | - | LS (MSH6) | - |
| Colon carcinoma | Isolated loss MSH6 | MSI (5) | Group 1 | - | LS (MSH6) | - |
| Rectal carcinoma | Loss of 4 proteins | MSI (5) | Group 4 | - | No genetic investigation | - |
| Colon carcinoma | Loss of 4 proteins | MSI (5) | Group 4 | - | LS (MLH1) | - |
| Colon carcinoma | Loss of 4 proteins | MSI (5) | Group 4 | - | No LS | - |
| Colon carcinoma | Loss MLH1/PMS2 | MSS | Group 2 | No NGS  MLH1 promotor methylation | No genetic investigation | - |
| Colon carcinoma | Isolated loss PMS2 | MSI (5) | Group 1 | - | LS (PMS2) | - |
| Colon carcinoma | Isolated loss PMS2 | MSI (5) | Group 1 | - | No genetic investigation | - |
| Colon carcinoma | Isolated loss MSH6 | MSI (4) | Group 1 | - | LS (MSH6) | - |
| Colon carcinoma | Isolated loss PMS2 | MSS | Group 1 | - | Genetic analysis on going | - |
| Colon carcinoma | Isolated loss MSH6 | MSI (4) | Group 1 | - | Genetic analysis on going | - |
| Colon carcinoma | Isolated loss MSH6 | MSI (3) | Group 1 | - | No genetic investigation | - |
| Colon carcinoma | Loss PMS2 - MSH6 | MSI (5) | Group 4 | - | Genetic analysis on going | - |
| Colon carcinoma | Retained 4 proteins | MSI (4) | Group 3 | - | LS (PMS2) | - |
| Colon carcinoma | Loss MLH1/PMS2 | MSS | Group 2 | No NGS  No methylation analysis | No genetic investigation | ICI |
| Colon carcinoma | Retained 4 proteins | MSI Low (1) | Group 3 | - | No genetic investigation | - |
| Colon carcinoma | Loss PMS2/MLH1 | MSI Low (1) | Group 2 | No NGS  MLH1 promotor methylation | No genetic investigation | - |
| Duodenal carcinoma | Isolated loss PMS2 | MSI (5) | Group 1 | MSI High  TMB High: 32  PMS2+MSH6 | No LS (no MMR germline mutation) | - |
| Duodenal carcinoma | Isolated loss PMS2 | MSI (5) | Group 1 | MSI High  TMB High: 52  PMS2 | LS (PMS2) | - |
| Gastric carcinoma | Loss of 4 MMR proteins | MSI (5) | Group 4 | MSI High  TMB High: 24  MSH2 + MSH6 | Not performed (dead) | - |
| Small bowel carcinoma | Loss MLH1/PMS2 | MSS | Group 2 | MSS  TMB low : 4  No mutation | No genetic investigation | - |
| Cholangio-carcinoma | Loss MLH1/PMS2 | MSS | Group 2 | - | Lynch (MLH1) | ICI |
| Gastric carcinoma | Isolated loss MSH6 | MSS | Group 1 | - | Genetic analysis on going | ICI |
| Gastric carcinoma | Isolated loss PMS2 | MSI (4) | Group 1 | - | No LS | - |
| Peritoneal mucinous carcinoma | Isolated loss MSH2 | MSI (5) | Group 1 | - | LS (MSH2) | ICI |
| Endometrial carcinoma | Loss MLH1/PMS2 | MSI Low (1) | Group 2 | MSS TMB intermediate: 13  No MMR mutation  Absence of MLH1 methylation | No genetic investigation | - |
| Endometrial carcinoma | Clonal loss MSH2/ MSH6 | MSI (5) | Group 4 | MSI High  TMB High: 501  POLD1 | LS (MSH2) | - |
| Endometrial carcinoma | Isolated loss MSH6 | MSI (3) | Group 1 | MSI High  TMB High: 28  MSH6 | Genetic analysis on going | - |
| Endometrial carcinoma | Isolated loss PMS2 | MSI (3) | Group 1 | - | Lynch PMS2 | - |
| Endometrial carcinoma | Isolated loss PMS2 | MSS | Group 1 | - | Genetic analysis on going | - |
| Endometrial carcinoma | Isolated loss MSH6 | MSI (2) | Group 1 | - | No genetic investigation | - |
| Endometrial carcinoma | Retained 4 MMR proteins | MSI Low (1) | Group 3 | - | No genetic investigation | - |
| Endometrial carcinoma | Loss PMS2/MSH6 | MSI Low (1) | Group 2 | - | LS (PMS2) | - |
| Endometrial carcinoma | Loss MLH1/PMS2 | MSS | Group 2 | No NGS  MLH1 promotor methylation | No genetic investigation | RT |
| Endometrial carcinoma | Loss MSH2/MSH6 | MSI Low (1) | Group 2 | - | Lynch MSH2 | - |
| Urothelial carcinoma | Isolated loss MSH6 | MSI (3) | Group 1 | MSS  TMB High: 37  Mutation MSH6 | LS (MSH6) | - |
| Sebaceous tumor | Loss MSH2/MSH6 | MSS | Group 2 | - | LS (MSH2) | - |
| Ovarian carcinoma | Isolated loss MSH6 | MSS | Group 1 | - | LS (MSH6) | - |
| Sebaceous tumor | Isolated loss MSH6 | MSS | Group 1 | - | No genetic investigation | - |
| Sebaceous tumor | Loss MSH2/MSH6 | MSI Low (1) | Group 2 | - | No genetic investigation | - |
| Sebaceous tumor | Loss MLH1/PMS2 | MSS | Group 2 | - | LS (MLH1) | - |
| Glioma | Loss MLH1 and MSH6, retained MSH2,  PMS2 NI | MSS | Group 4 | - | LS (MLH1) | - |
| Ovarian carcinoma | Isolated loss PMS2 | MSS | Group 1 | - | LS (PMS2) | - |
| Sebaceous tumor | Loss MSH2/MSH6 | MSI Low (1) | Group 2 | - | LS (MSH2) | - |
| Sebaceous tumor | Isolated loss MSH6 | MSS | Group 1 | - | No genetic investigation | - |
| Sarcoma | Loss 4 MMR proteins | MSS | Group 4 | - | LS (MLH1) | RCT |

The MSI-PCR result is given as MSI-High, MSS or MSI-Low, followed in parenthesis by the number of unstable microsatellites from the five MSI-PCR markers. The NGS results include the MSI status (classified as MSS or MSI-High), the tumor mutation burden (TMB) (classified as TMB-high, -intermediate or -low) followed by the value of the TMB (mutations/Mb) and relevant somatic mutations (MMR or POL mutations) when identified in the tumor. MLH1 promotor methylation analysis is presented as: not analyzed, no methylation or methylation. Results for genetics are reported as the type of genetic predisposition (LS: Lynch syndrome, CMMRD: constitutional MMR deficiency, followed in parenthesis by the germinal mutation) when identified. Only relevant treatments for this study are reported (neoadjuvant therapy and immune checkpoint inhibitor). The gray lines correspond to the tumor for which the origin of MMR inactivation could not be identified.

MMR: mismatch repair. MSI: microsatellite instability. MMR-D: mismatch repair- deficiency. TMB: tumor mutation burden. NI: not interpretable. RT: radiotherapy. RCT: radiochemotherapy. ICI : immune checkpoint inhibitor

**Table S2 (online only):** Impact of the type of MMR protein inactivation on unusual MMR-D phenotype.

|  | MSI-High | MSI-Low | MSS | All |
| --- | --- | --- | --- | --- |
| **PMS2 inactivation** | **20** | **1** | **4** | **25** |
| Mutation (germinal or somatic)  Isolated PMS2 loss  PMS2/MLH1 Loss  PMS2 retained  Other profiles | 12 (7+5)  0  1  3* | 0  0  0  1** | 2  0  0  0 | **19**  14  0  1  4 |
| No Mutation or no germinal or somatic investigations  Isolated PMS2 loss  PMS2/MLH1 Loss  PMS2 retained  Other profiles | 3  0  0  1*** | 0  0  0  0 | 2  0  0  0 | **6**  5  0  0  1 |
| **MSH6 inactivation** | **27** | **1** | **6** | **34** |
| Mutation (germinal or somatic)  Isolated MSH6 loss  MSH6/MSH2 Loss  MSH6 retained  Other profiles*** | 17 (8+9)  0  0  1 | 0  0  0  0 | 2 (1+1)  0  0  0 | **20**  19  0  0  1 |
| No Mutation or no germinal or somatic investigations  Isolated MSH6 loss  MSH6/MSH2 Loss  MSH6 retained  Other profiles | 9  0  0  0 | 1  0 0  0 | 4  0  0  0 | **14**  14  0  0  0 |
| **MLH1 inactivation** | **4** | **4** | **7** | **15** |
| Mutation (germinal or somatic)  Isolated MLH1 loss  MLH1/PMS2 Loss  MLH1 retained  Other profiles**** | 0  0  0  4 | 0  1  0  0 | 0  4 (2+2)  0  1 | **10**  0  5  0  5 |
| No Mutation or no germinal or somatic investigations  Isolated MLH1 loss  MLH1/PMS2 Loss  MLH1 retained  Other profiles | 0  0  0  0 | 0  3  0  0 | 0  2  0  0 | **5**  0  5  0  0 |
| **MSH2 inactivation** | **3** | **3** | **2** | **8** |
| Mutation (germinal or somatic)  Isolated MSH2 loss  MSH2/MSH6 Loss  MSH2 retained  Other profiles***** | 1  0  1  1 | 0  2  0  0 | 0  1  0  0 | **6**  1  3  1  1 |
| No Mutation or no germinal or somatic investigations  Isolated MSH2 loss  MSH2/MSH6 Loss  MSH2 retained  Other profiles | 0  0  0  0 | 0  1  0  0 | 0  1  0  0 | **2**  0  2  0  0 |
| **All inactivated proteins** | 54 | 9 | 19 | **82** |

*: Other profiles corresponded to one clonal loss of PMS2/MLH1, one clonal loss of PMS2 only, one PMS2 staining not interpretable and one PMS2/MSH6 loss.

**: This case was a PMS2 staining that was not interpretable

***: This case was a MSH6 staining that was not interpretable

****: Other profiles corresponded to one MLH1 retained with PMS2 not interpretable, one clonal loss of MLH1/PMS2, two losses of 4 MMR proteins, one loss of MLH1 with PMS2 not interpretable

*****: This case was a clonal loss of MSH2/MSH6

In line “Mutation”, the underlined number corresponds to cases with germinal mutation, and the number not underlined to somatic mutation.

Two kinds of inactivation of each MMR protein were defined: one category was defined by the identification of a germinal or somatic mutation of the corresponding gene (based on genetic or tumor NGS analysis or on MLH1 promotor methylation in the tumor) and another was defined by loss of expression of one MMR protein but with no mutation identified (neither germinal nor somatic) or no genetic investigations. For each category, the IHC profile and MSI status were reported. Six cases failed to be classified because the type of inactivated protein was not identified (3 cases corresponded to retained staining of the four MMR proteins with MSI but no genetic investigation and 3 cases to loss of the four MMR proteins with no mutation or no genetic investigation). Another case corresponded to a POLE mutation. Overall, 82 cases could be classified.
